# FDA Escherichia coli Identification (FDA-ECID) Microarray: a Pangenome Molecular Toolbox for Serotyping, Virulence Profiling, Molecular Epidemiology, and Phylogeny

**DOI:** 10.1128/AEM.04077-15

**Published:** 2016-05-16

**Authors:** Isha R. Patel, Jayanthi Gangiredla, David W. Lacher, Mark K. Mammel, Scott A. Jackson, Keith A. Lampel, Christopher A. Elkins

**Affiliations:** Division of Molecular Biology, Office of Applied Research and Safety Assessment, Center for Food Safety and Applied Nutrition, U.S. Food and Drug Administration, Laurel, Maryland, USA; Washington State University

## Abstract

Most Escherichia coli strains are nonpathogenic. However, for clinical diagnosis and food safety analysis, current identification methods for pathogenic E. coli either are time-consuming and/or provide limited information. Here, we utilized a custom DNA microarray with informative genetic features extracted from 368 sequence sets for rapid and high-throughput pathogen identification. The FDA Escherichia coli Identification (FDA-ECID) platform contains three sets of molecularly informative features that together stratify strain identification and relatedness. First, 53 known flagellin alleles, 103 alleles of *wzx* and *wzy*, and 5 alleles of *wzm* provide molecular serotyping utility. Second, 41,932 probe sets representing the pan-genome of E. coli provide strain-level gene content information. Third, approximately 125,000 single nucleotide polymorphisms (SNPs) of available whole-genome sequences (WGS) were distilled to 9,984 SNPs capable of recapitulating the E. coli phylogeny. We analyzed 103 diverse E. coli strains with available WGS data, including those associated with past foodborne illnesses, to determine robustness and accuracy. The array was able to accurately identify the molecular O and H serotypes, potentially correcting serological failures and providing better resolution for H-nontypeable/nonmotile phenotypes. In addition, molecular risk assessment was possible with key virulence marker identifications. Epidemiologically, each strain had a unique comparative genomic fingerprint that was extended to an additional 507 food and clinical isolates. Finally, a 99.7% phylogenetic concordance was established between microarray analysis and WGS using SNP-level data for advanced genome typing. Our study demonstrates FDA-ECID as a powerful tool for epidemiology and molecular risk assessment with the capacity to profile the global landscape and diversity of E. coli.

**IMPORTANCE** This study describes a robust, state-of-the-art platform developed from available whole-genome sequences of E. coli and Shigella spp. by distilling useful signatures for epidemiology and molecular risk assessment into one assay. The FDA-ECID microarray contains features that enable comprehensive molecular serotyping and virulence profiling along with genome-scale genotyping and SNP analysis. Hence, it is a molecular toolbox that stratifies strain identification and pathogenic potential in the contexts of epidemiology and phylogeny. We applied this tool to strains from food, environmental, and clinical sources, resulting in significantly greater phylogenetic and strain-specific resolution than previously reported for available typing methods.

## INTRODUCTION

*Escherichia* spp. are Gram-negative, facultative anaerobes belonging to the Enterobacteriaceae family. Most E. coli strains are commensals and are found as part of the gut microbiota, benefitting their hosts by producing essential compounds, such as vitamin K2, and also by establishing a “colonization barrier effect” to prevent the invasion of pathogenic bacteria into intestinal cells. Long-standing phylogenetic analyses of E. coli suggest it can be divided, by the latest measure, into at least seven phylogroups: A, B1, B2, C, D, E, and F (1–3). In addition, five cryptic lineages of Escherichia that are phenotypically indistinguishable from E. coli under standard microbiological assays have been reported (4).

Pathogenic E. coli strains have evolved to adapt to humans as a host, and in some cases they colonize animal species asymptomatically; collectively they are intercalated across the phylogroups. The pathogenic E. coli serotypes have been further subdivided according to their typical sites of infection and clinical manifestations in humans (5, 6). A common means to identify pathogenic E. coli usually involves tests for major serotypes with a history of disease and key genetic virulence markers. As an example, Shiga toxin-producing E. coli (STEC) strains are capable of expressing Shiga toxin type 1 (Stx1) and/or type 2 (Stx2), potent cytotoxins encoded by the *stx*_1_ and *stx*_2_ genes, respectively. STEC strains that cause hemolytic uremic syndrome (HUS) in humans more often produce Stx2, and there are several subtypes of *stx*_2_ that are responsible for differences in cytotoxicity (7–9). Additionally, STEC strains carry other virulence factors such as the intimin adhesin, an outer membrane protein essential for the formation of the characteristic attaching-and-effacing (A/E) lesion of enteropathogenic E. coli (EPEC) and enterohemorrhagic E. coli (EHEC) strains (10, 11). The 94-kDa intimin protein is encoded by the *eae* gene located on the locus of enterocyte effacement (LEE) pathogenicity island (12). This gene is highly polymorphic, with over 25 major allelic variants being reported (13). Another putative virulence marker is the plasmid-borne enterohemolysin gene (*ehxA*), which has been found in EHEC and STEC strains (14, 15) and has been used as an epidemiological marker in pathogenic strains (16, 17). The combination of overall genome content and virulence factors is variable in STEC (18). Indeed, there is a limited correlation between serotype and strains with pathogenic potential to cause human illness, whereas an accurate detection of *stx* subtypes, *eae*, and other virulence factors is a better indicator of virulence.

In the United States, foodborne illnesses affect about 48 million people annually (19). According to the CDC's public health surveillance system, the bacteria most often implicated in foodborne diseases are Campylobacter, Listeria, Salmonella, STEC, Shigella, Vibrio, and Yersinia. As the prototypic EHEC serogroup, E. coli O157:H7 is a formidable pathogen; however, other serogroups, i.e., non-O157 STEC, have been implicated in several foodborne outbreaks, notably in fresh produce (20). In addition, STEC strains are commonly found in food, yet the ability to detect and identify these microbes in foods is challenging (21). Efforts to develop effective preventive measures, as well as rapid methods to identify these pathogens for outbreak response or surveillance programs, are critical components for public health safety.

A rapid, specific diagnostic test to distinguish pathogenic and nonpathogenic E. coli in food analytical laboratories has great practical value to prevent and respond to foodborne outbreaks. In this study, we exploited the rapidly evolving whole-genome sequence (WGS) technology and used 368 publically available E. coli and Shigella sequence sets to design the Food and Drug Administration Escherichia coli Identification (FDA-ECID) microarray. In a similar fashion, we previously designed and used a multigenome custom microarray to assess the total gene content of pathogenic E. coli in the 2011 O104:H4 outbreak (22). The basis for these initial efforts involved whole-genome genotyping of E. coli and was rooted in genomic discovery, but interestingly, in retrospect, it provided intrinsic epidemiological and phylogenetic signatures for strain-level discrimination. We expanded this concept here in the WGS era from basic gene content to incorporate molecular serotype and virulence determination with deep phylogenetic profiling of individual strains using WGS single nucleotide polymorphism (SNP)-level discrimination. This molecular toolbox can accurately resolve and stratify identification without a comparative reference using unique probe set design analysis strategies.

## MATERIALS AND METHODS

### Bacterial strains.

A total of 610 isolates were examined in this study (see Table S1 in the supplemental material). This diverse collection of strains was selected to represent the range of genotypic variation within the species and includes both the E. coli reference (ECOR) and diarrheagenic E. coli (DEC) collections. All strains were grown in 3 ml of Luria broth and incubated overnight at 37°C with moderate shaking. A subset of 103 strains was selected for more in-depth analysis because of the availability of WGS data (Table 1).

**TABLE 1:** Strains investigated with the FDA-ECID microarray and whole-genome sequencing

Strain	Group^*a*^	GenBank ID	Other designation(s)	Serotype^*b*^						*stx*_1_		*stx*_2_		*eae*		*ehxA*	
				O			H										
				Reported	ECID	WGS	Reported	ECID	WGS	ECID	WGS	ECID	WGS	ECID	WGS	ECID	WGS
K-12	A	U00096	MG1655	Rough	16	16	48	48	48	−	−	−	−	−	−	−	−
EC1439	A	AIFV	DEC 6A	111	111	111	12	12	12	−	−	−	−	−	−	−	−
EC1440	A	AIFW	DEC 6B	111	111	111	12	12	12	−	−	−	−	−	−	−	−
EC1441	A	AIFX	DEC 6C	111	111	111	12	12	12	−	−	−	−	−	−	−	−
EC1442	A	AIFY	DEC 6D	111	111	111	4	4	4	−	−	−	−	−	−	−	−
EC1443	A	AIFZ	DEC 6E	111	111	111	NM	4	4	−	−	−	−	−	−	−	−
EC1445	A	AIGB	DEC 7B	149	157	157	NM	42	42	−	−	−	−	−	−	−	−
EC1444	B1	AIGA	DEC 7A	157	157	157	43	43	43	−	−	−	−	−	−	−	−
EC1446	B1	AIGC	DEC 7C	157	157	157	43	43	43	−	−	−	−	−	−	−	−
EC1447	B1	AIGD	DEC 7D	157	157	157	43	43	43	−	−	−	−	−	−	−	−
EC1448	B1	AIGE	DEC 7E	157	157	157	NM	43	43	−	−	−	−	−	−	−	−
EC1449	B1	AIGF	DEC 8A	111	111	111	NM	8	8	a	a	−	−	γ2	γ2	+	+
EC1450	B1	AIGG	DEC 8B	111	111	111	8	8	8	a	a	a/c/d	a	γ2	γ2	+	+
EC1451	B1	AIGH	DEC 8C	111	111	111	NM	11	11	a	a	−	−	β1	β1	+	+
EC1452	B1	AIGI	DEC 8D	111	111	111	11	11	11	−	−	−	−	β1	β1	+	+
EC1453	B1	AIGJ	DEC 8E	111	111	111	8	8	8	a	a	−	−	γ2	γ2	−	−
EC1454	B1	AIGK	DEC 9A	26	26	26	11	11	11	−	−	−	−	β1	β1	−	−
EC1455	B1	AIGL	DEC 9B	26	26	26	NM	11	11	−	−	−	−	β1	β1	−	−
EC1456	B1	AIGM	DEC 9C	26	26	26	NM	11	11	−	−	−	−	β1	β1	−	−
EC1457	B1	AIGN	DEC 9D	26	26	26	11	11	11	−	−	−	−	β1	β1	−	−
EC1458	B1	AIGO	DEC 9E	26	26	26	11	11	11	−	−	−	−	β1	β1	−	−
EC1459	B1	AIGP	DEC 10A	26	26	26	11	11	11	a	a	−	−	β1	β1	+	−/+^*d*^
EC1460	B1	AIGQ	DEC 10B	26	26	26	11	11	11	a	a	−	−	β1	β1	+	−/+
EC1461	B1	AIGR	DEC 10C	26	26	26	11	11	11	a	a	−	−	β1	β1	−	−
EC1462	B1	AIGS	DEC 10D	26	26	26	11	11	11	−	−	−	−	β1	β1	+	+
EC1463	B1	AFAI	DEC 10E	26	26	26	11	11	11	a	a	−	−	β1	β1	+	−
EC1464	B1	AIGU	DEC 10F (RDEC-1)	15	15	15	NM	11	11	−	−	−	−	β1	β1	−	−
EC1465	B1	AIGV	DEC 11A	128	128	128	2	2	2	−	−	−	−	β1	β1	−	−
EC1466	B1	AIGW	DEC 11B	128	128	128	2	2	2	−	−	−	−	β1	β1	−	−
EC1467	B1	AIGX	DEC 11C	45	45	45	2	2	2	a	a	−	−	ε1	ε1	+	+
EC1468	B1	AIGY	DEC 11D	128	128	128	2	2	2	−	−	−	−	β1	β1	−	−
EC1469	B1	AIGZ	DEC 11E	128	128	128	2	2	2	−	−	−	−	β1	β1	−	−
EC1470	B1	AIHA	DEC 12A	111	111	111	2	2	2	−	−	−	−	β1	β1	−	−
EC1471	B1	AIHB	DEC 12B	111	111	111	2	2	2	−	−	−	−	β1	β1	−	−
EC1472	B1	AIHC	DEC 12C	111	111	111	NM	2	2	−	−	−	−	β1	β1	−	−
EC1473	B1	AIHD	DEC 12D	111	111	111	2	2	2	−	−	−	−	β1	β1	−	−
EC1474	B1	AIHE	DEC 12E	111	111	111	NM	2	2	−	−	−	−	β1	β1	−	−
EC1375	B1	AAJX	DEC 12F (B171)	111	111	111	NM	2	2	−	−	−	−	β1	β1	−	−
EC1475	B1	AIHF	DEC 13A	128	128	128	7	7	7	−	−	−	−	−	−	−	−
EC1476	B1	AIHG	DEC 13B	128	128	128	7	7	7	−	−	−	−	−	−	−	−
EC1477	B1	AIHH	DEC 13C	128	128	128	7	7	7	−	−	−	−	−	−	−	−
EC1478	B1	AIHI	DEC 13D	128	128	128	7	7	7	−	−	−	−	−	−	−	−
EC1479	B1	AIHJ	DEC 13E	128	128	128	47	7	7	−	−	−	−	−	−	−	−
EC1480	B1	AIHK	DEC 14A	128	86	86	21	8	8	−	−	−	−	−	−	−	−
EC1481	B1	AIHL	DEC 14B	128	128	128	21	21	21	−	−	−	−	−	−	−	−
EC1482	B1	AIHM	DEC 14C	128	128	128	21	21	21	−	−	−	−	−	−	−	−
EC1483	B1	AIHN	DEC 14D	128	128	128	NM	21	21	−	−	−	−	−	−	−	−
EC1484	B1	AIGT	DEC 14E	128	128	128	21	21	21	−	−	−	−	−	−	−	−
EC1485	B1	AIHO	DEC 15A	111	111	111	21	21	21	−	−	−	−	−	−	−	−
EC1486	B1	AIHP	DEC 15B	111	111	111	21	21	21	−	−	−	−	−	−	−	−
EC1487	B1	AIHQ	DEC 15C	111	111	111	21	21	21	−	−	−	−	−	−	−	−
EC1488	B1	AIHR	DEC 15D	111	111	111	21	21	21	−	−	−	−	−	−	−	−
EC1489	B1	AIHS	DEC 15E	111	111	111	21	21	21	−	−	−	−	−	−	−	−
EC1514	B1	CP000800	E24377A	139	NT	139v^*c*^	28	28	28	−	−	−	−	−	−	−	−
EC1517	B1	AAJW	E110019	111	111	111	9	9	9	−	−	−	−	α4	α4	−	−
EC1518	B1	AAJV	E22	103	103	103	2	2	2	−	−	−	−	β1	β1	−	−
EC1520	B1	CP005998	B7A	148	148	148	28	28	28	−	−	−	−	−	−	−	−
EC1892	B1	CP003297	2009EL-2050	104	104	104	4	4	4	−	−	a/c/d	a	−	−	−	−
EC1893	B1	CP003301	2009EL-2071	104	104	104	4	4	4	−	−	a/c/d	a	−	−	−	−
EC1894	B1	CP003289	2011C-3493	104	104	104	4	4	4	−	−	a/c/d	a	−	−	−	−
EC1908	B1	CU928145	55989	104	104	104	4	4	4	−	−	−	−	−	−	−	−
EC1412	B2	AIEV	DEC 1A	55	55	55	6	6	6	−	−	−	−	α1	α1	−	−
EC1413	B2	AIEW	DEC 1B	55	55	55	6	6	6	−	−	−	−	α1	α1	−	−
EC1414	B2	AIEX	DEC 1C	55	55	55	6	6	6	−	−	−	−	α1	α1	−	−
EC1415	B2	AIEY	DEC 1D	55	55	55	6	6	6	−	−	−	−	α1	α1	−	−
EC1416	B2	AIEZ	DEC 1E	55	55	55	6	6	6	−	−	−	−	α1	α1	−	−
EC1417	B2	AIFA	DEC 2A	55	55	55	6	6	6	−	−	−	−	α1	α1	−	−
EC1418	B2	AFJB	DEC 2B	55	55	55	NM	6	6	−	−	−	−	α1	α1	−	−
EC1419	B2	AIFB	DEC 2C	55	55	55	6	6	6	−	−	−	−	α1	α1	−	−
EC1420	B2	AIFC	DEC 2D	55	55	55	6	6	6	−	−	−	−	α1	α1	−	−
EC1421	B2	AIFD	DEC 2E	55	55	55	6	6	6	−	−	−	−	α1	α1	−	−
EC1519	B2	AAJU	F11	6	6	6	31	31	31	−	−	−	−	−	−	−	−
EC1521	B2	AE014075	CFT073	6	6	6	1	1	1	−	−	−	−	−	−	−	−
EC1274	E	CP008957	EDL933	157	157	157	7	7	7	a	a	a/c/d	a	γ1	γ1	+	+
EC1276	E	BA000007	Sakai	157	157	157	7	7	7	a	a	a/c/d	a	γ1	γ1	+	+
EC1422	E	AIFE	DEC 3A	157	157	157	7	7	7	a	a	a/c/d	a	γ1	γ1	+	+
EC1423	E	AIFF	DEC 3B	157	157	157	7	7	7	a	a	a/c/d	a	γ1	γ1	+	+
EC1424	E	AIFG	DEC 3C	157	157	157	7	7	7	a	a	a/c/d	a	γ1	γ1	+	+
EC1425	E	AIFH	DEC 3D	157	157	157	7	7	7	a	a	a/c/d	a	γ1	γ1	+	+
EC1426	E	AIFI	DEC 3E	157	157	157	7	7	7	−	−	a/c/d	c	γ1	γ1	+	+
EC1427	E	AIFJ	DEC 3F (493/89)	157	157	157	NM	7	7	−	−	a/c/d	a	γ1	γ1	+	+
EC1428	E	AIFK	DEC 4A	157	157	157	7	7	7	−	−	−	−	γ1	γ1	+	+
EC1429	E	AIFL	DEC 4B	157	157	157	7	7	7	−	−	a/c/d	a, c	γ1	γ1	+	+
EC1430	E	AIFM	DEC 4C	157	157	157	7	7	7	−	−	−	−	γ1	γ1	+	+
EC1431	E	AIFN	DEC 4D	157	157	157	7	7	7	−	−	a/c/d	c	γ1	γ1	+	+
EC1432	E	AIFO	DEC 4E	157	157	157	7	7	7	a	a	−	−	γ1	γ1	+	+
EC1433	E	AIFP	DEC 4F (EDL933)	157	157	157	7	7	7	a	a	a/c/d	a	γ1	γ1	+	+
EC1434	E	AIFQ	DEC 5A	55	55	55	7	7	7	−	−	−	−	γ1	γ1	−	−
EC1435	E	AIFR	DEC 5B	55	55	55	7	7	7	−	−	−	−	γ1	γ1	−	−
EC1436	E	AIFS	DEC 5C	55	55	55	7	7	7	−	−	−	−	γ1	γ1	−	−
EC1437	E	AIFT	DEC 5D	55	55	55	7	7	7	−	−	−	−	γ1	γ1	−	−
EC1438	E	AIFU	DEC 5E	55	55	55	7	7	7	−	−	−	−	γ1	γ1	−	−
EC1734	E	AKMO	09PF 532	157	157	157	7	7	7	−	−	a/c/d	a	γ1	γ1	+	+
EC1738	E	AKMN	550659	157	157	157	7	7	7	−	−	a/c/d	c	γ1	γ1	+	+
EC4045	E	ABHL	FD 888 C1	157	157	157	7	7	7	−	−	a/c/d	a, c	γ1	γ1	+	+
EC4076	E	ABHQ	BAC0600006766	157	157	157	7	7	7	−	−	a/c/d	a, c	γ1	γ1	+	+
EC4115	E	CP001164	06MMIS0960	157	157	157	7	7	7	−	−	a/c/d	a, c	γ1	γ1	+	+
EC4206	E	ABHK	06X04242	157	157	157	7	7	7	−	−	a/c/d	a, c	γ1	γ1	+	+
EC4401	E	ABHR	06E02109	157	157	157	7	7	7	−	−	a/c/d	a, c	γ1	γ1	+	+
EC2822	CL1	AEJX	TW15838	NA	2	2	NA	45	45	a	a	a/c/d	a	−	−	+	+
EC2817	CL3	AEJW	TW09231	NA	NT	10-like^*c*^	NA	52	52	−	−	−	−	−	−	−	−
EC2819	CL4	AEMF	TW11588	NA	NT	36-like^*c*^	NA	NT	5/56-like^*c*^	−	−	−	−	−	−	−	−
EC2818	CL5	AEME	TW09308	NA	NT	139v-like^*c*^	NA	56	56	−	−	−	−	−	−	−	−

a E. coli phylogroup or cryptic lineage (CL).

b NA, not available; NM, nonmotile; NT, nontypeable.

c Not represented on the array.

d Negative in the WGS contigs but positive in the SRA reads.

### Microarray design.

A total of 368 E. coli and Shigella sequence sets were used to identify 55,918 annotated open reading frames, from which 41,932 probe sets were selected using Affymetrix's probe set design software (Affymetrix, Santa Clara, CA). The sequence sets include 54 closed chromosomes, 47 closed plasmids, and 267 whole-genome shotgun sequences from GenBank (see Table S2 in the supplemental material). For each targeted genomic region, the design strategy created a probe set comprising, on average, 11 probe pairs. Each probe pair consists of one 25-mer oligomer that matches the reference sequence and a corresponding mismatch 25-mer that differs from the perfect match by a single nucleotide at the central (13th) position of the oligonucleotide (23). The probe set signal is the summation of the 11 individual probe pair signals in which the mismatch probe signal is used to correct for nonspecific hybridization.

We included 211 unique probe sets for identifying 152 O types and 54 probe sets for all known H types. Additionally, four and eight probe sets were included for the detection and/or allelic subtyping of *stx*_1_ and *stx*_2_, respectively. Where possible, probe sets were named with the GenBank reference sequence that was used for their design (see, e.g., *stx* in Table S3 in the supplemental material). Detection and subtyping of *eae* were accomplished using 48 probe sets for different regions of this highly diverse locus. DNA sequence similarity in the 3′ half of the *eae* gene, which corresponds to the extracellular domains of the intimin protein, was used to organize the probe sets into seven allele families: α (11 alleles), β (5 alleles), γ (8 alleles), ε (8 alleles), ι (3 alleles), λ (4 alleles), and ρ (3 alleles) (see Table S4 in the supplemental material). In order to detect any novel *eae* alleles not represented by the probe sets targeting the extracellular domains, we also included probe sets for the conserved transmembrane domain of the intimin protein. Probe sets for the detection of other virulence genes are included as part of the pan-genome.

Using the same 321 chromosomal members of the reference sequence sets, we identified ∼125,000 conserved 25-mers, each containing a central single nucleotide polymorphism (SNP). Of these, we filtered the 10% most informative SNP sites by favoring SNPs which give a unique pattern of change over the 321 chromosomal sequences. Each of the 9,984 discriminatory SNP sites is based on the reference genome for K-12 MG1655 (GenBank accession number U00096.2) and is represented on the FDA-ECID microarray using an SNP-typing probe strategy.

### DNA isolation and microarray hybridization.

Total genomic DNA was extracted from 1 ml of culture using the Qiagen DNeasy kit. DNA extractions were performed with the Qiagen QIAcube instrument using the protocol for isolation of DNA from Gram-negative bacteria (Qiagen, Hilden, Germany). The eluted DNA was further purified and concentrated using Amicon Ultra-0.5 30K filters (Merck KgaA, Darmstadt, Germany). In order to improve the efficiency of hybridization of the target DNA to the microarray, DNA was randomly fragmented to an average molecular size of ∼200 bp by DNase I digestion. Briefly, each DNA sample (2 μg) was digested with 0.01 unit of RQ1 RNase-free DNase I (Promega, Sunnyvale, CA) at 37°C for 1 min, which was immediately followed by incubation at 99°C for 15 min to denature the DNase I. The digested DNA was then 3′-end labeled with biotin-11-ddATP (PerkinElmer, Akron, OH) using 30 units of recombinant terminal deoxynucleotidyl transferase (rTdT) (Affymetrix) and incubated at 37°C for 3 h. Next, 35 μl of 1.3× HybA, 65.8 μl of HybB, and 2.2 μl of B2 Oligo Control from the GeneChip GeneAtlas hybridization and stain kit (Affymetrix) were added to the labeled DNA, which was then incubated at 96°C for 10 min to denature the DNA and then cooled to 45°C for 2 min. To each of the four wells of the GeneAtlas hybridization tray, 120 μl of respective denatured sample was added, and the FDA-ECID array strip (with four arrays) was placed on the hybridization tray and incubated at 45°C for 16 h. Following hybridization, the arrays were washed and scanned using the Affymetrix GeneAtlas system according to the default settings in the GeneAtlas instrument control software.

### Gene-level probe set data summarization.

The Robust MultiArray Averaging (RMA) function in the affy package of R-Bioconductor was utilized to carry out background subtractions, normalizations, and probe set summarizations, in batch, on the array-generated (.cel) data files (24, 25). The RMA summarized values were then used to perform hierarchical cluster (HC) analysis using average linkage clustering with a Pearson correlation measure of similarity. The MAS5.0 algorithm was also used by changing the default parameters to τ = 0.15, α1 = 0.05, and α2 = 0.05 (the custom R script can be provided upon request) (23). MAS5 calls were useful in determining discrete individual gene presence or absence (sequence divergence) for molecular serotyping and virulence typing. HC analyses were performed using the number of probe sets greater than 3-fold different in their RMA intensity values. The reproducibility of the array was verified through triplicate runs of four reference strains (data not shown).

### SNP data summarization.

Affymetrix's GeneChip Sequence (GSEQ) analysis software was used to batch analyze the .cel files for the 103 strains with available WGS data, determining if each SNP on the array was a match to the reference sequence. A quality score was assigned to each position based on the respective hybridization intensity for both forward- and reverse-strand probes. Base calls and quality scores were determined using the haploid model system with a base reliability threshold of 0.5 and a quality score threshold of 1, respectively. Optimal threshold values for the base reliability and quality scores were determined by using WGS data for the same SNPs. For each data set (microarray and WGS), the 9,984 SNP sites were concatenated and neighbor-joining trees were constructed from a *p* distance matrix using MEGA (26).

## RESULTS

### Molecular serotyping.

The MAS5 calls (present or divergent) were used to determine the O and H types of the strains investigated. The reported serological serotype and the molecular serotype detected by the array as well as by WGS for the 103 strains with available WGS data are shown in Table 1. The O types determined via the array were in complete agreement with the WGS data. When comparing the traditional serological data to the molecular O serotypes determined by the array, three of the strains were identified with different O types by the array. As reported in our previous study (27), the molecular serotypes of strains DEC 7B and DEC 14A do not agree with their reported serological serotypes and are likely the result of the strains being either mislabeled or mistyped by serology. The third and final O-type inconsistency is the O rough phenotype of strain K-12 that was accurately typed as being O16 molecularly.

The serological serotypes for the strains that are from the cryptic lineages of Escherichia are not available, so comparisons between the two methodologies were not possible. The array was able to identify O types for 99 of the 103 strains, with the only O-nontypeable E. coli strain being E24377A because its O type is not represented on the array. Strain E24377A is reported to be O139:H28, but with the exception of the genes required for the dTDP-sugar biosynthesis pathway (*rmlBDAC*), the sequence of its O-antigen gene cluster is not homologous with that of the O-antigen gene cluster from the O139 type strain (GenBank accession no. DQ109552). Therefore, we refer to the variant O139 found in E24377A as O139v. The O types of the three remaining nontypeable strains (TW09231, TW09308, and TW11588) are also not represented on the array. Each of these three strains belongs to a different cryptic lineage of Escherichia, and the O-antigen gene clusters of strains TW09231, TW09308, and TW11588 are 93, 88, and 87% similar to O10, O139v, and O36, respectively, based on WGS data.

For the H types, 15 strains had differing results when comparing the array to traditional serology. The serological H types for 14 of these strains were unavailable due to nonmotility, whereas the molecular H types for all the E. coli strains were determined (Table 1). In comparison with WGS, the microarray correctly typed the molecular H types with the exception of the cryptic lineage 4 isolate TW11588. The WGS data show that this isolate carries a novel *fliC* allele that is approximately 90% homologous to the H5 and H56 *fliC* alleles represented on the array. Since this allele is outside the detectable limit of the array, strain TW11588 was classified as H nontypeable by the array. The O and H types identified via the array for the additional 507 strains in our database are listed in Table S1 in the supplemental material.

### Virulence profiling.

The RMA summarized intensities and *P* values were used to confirm the calls for the presence or absence of *stx*_1_, *stx*_2_, *eae*, and *ehxA* in each of the 103 strains examined (Table 1). Allelic variants of *stx*_1_ (*stx*_1a_, *stx*_1c_, and *stx*_1d_) can be accurately identified based on the MAS5 calls, but for *stx*_2_, the probe sets hybridized to multiple alleles because of the inherent mosaic nature of the *stx*_2_ subtypes attributable to recombination. Thus, while *stx*_2_ could be accurately detected by the array, allelic discrimination was more difficult. For the 103 strains examined, approximately 17% had *stx*_1_ and 22% had *stx*_2_. All of the *stx*_1_-positive strains examined possessed the *stx*_1a_ allele, which was in agreement with the WGS data (Table 1). For *stx*_2_, the array was 100% accurate both in making a present call and in suggesting the possible combination of alleles that could be present. For example, strain EC4045 had some combination of the *stx*_2a/c/d_ alleles based on the array, and WGS data reported a combination of *stx*_2a_ and *stx*_2c_.

Sixty-five strains (63%) were *eae* positive according to the array (Table 1). Of the seven *eae* allele families represented on the array, the γ family was most frequently observed among the 103 strains (45%), followed by β (37%), α (17%), and ε (1%). The remaining three families (ι, λ, and ρ) were not found in any of the strains in this study. Finally, 27% of the strains in this study showed presence of *ehxA*, with three strains being discrepant between the array and WGS data (Table 1). Two of these strains, DEC 10A and DEC 10B, were *ehxA* negative according to their WGS contig assemblies but were *ehxA* positive when their raw reads from the sequence read archive (SRA) were analyzed, suggesting that one or more assembly parameters may have led to the false-negative result in the WGS contig data. The remaining discrepant strain, DEC 10E, was *ehxA* negative in both the WGS and SRA data, suggesting that the sequenced isolate possibly lost the plasmid carrying this locus.

### Whole-genome genotyping.

The resulting tree from the RMA data conserves general phylogroup distribution and is useful for rapidly capturing unique gene content with appropriate reference comparison. However, the RMA data-based tree cannot be used to infer deeper phylogenetic relationships, since the gene content profiles are an amalgamation of core and mobile genetic elements that distort evolutionary relationships (see Fig. S1 in the supplemental material). Our analysis of the 610 strains includes isolates implicated in a few different outbreaks; examples include (i) O157:H7 isolates believed to be linked with the 2009 cookie dough-associated outbreak, (ii) clinical, bovine, and environmental O157:H7 isolates implicated in the 2006 spinach-associated outbreak, and (iii) O104:H4 isolates from the 2011 sprout-associated outbreak in Germany.

### (i) E. coli O157:H7 implicated in the 2009 cookie dough-associated outbreak.

**FIG 1:**
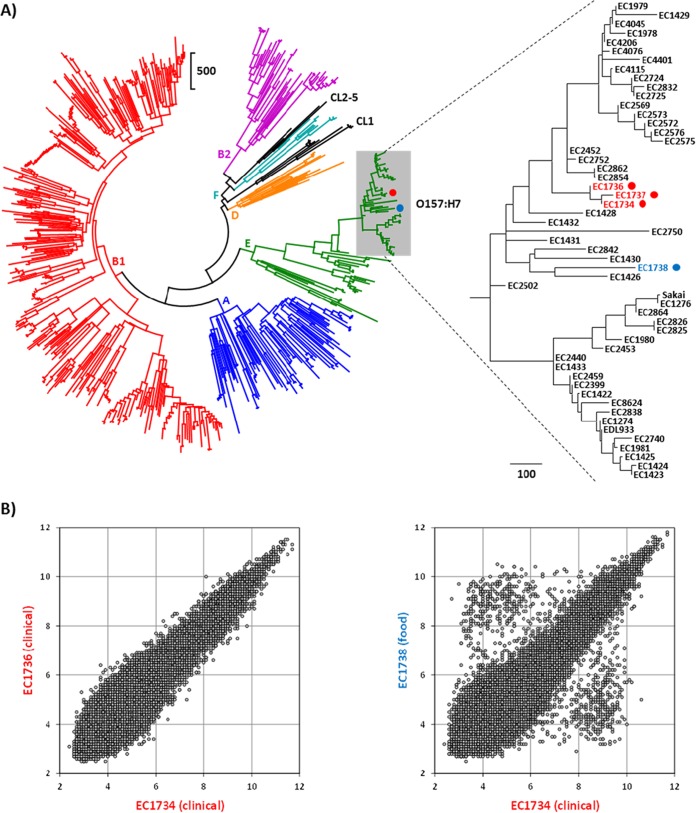
Genotype analysis of the O157:H7 strains implicated in the 2009 cookie dough-associated outbreak. (A) Hierarchical cluster dendrogram generated using the number of probe sets that were greater than 3-fold different in 610 strains. Scale bars represent the number of probe set differences, and O157:H7 strains are enclosed by the gray box. Clinical and food strains from the outbreak are indicated by the red and blue circles, respectively. (B) Scatter plots generated using the RMA intensities from all 41,932 probe sets for comparing two clinical isolates (left) and a clinical isolate and a food isolate (right).

To determine whether a strain being tested has been previously observed in our database, hierarchical cluster (HC) analysis was performed on all strains investigated to date using the number of probe sets greater than 3-fold different in their RMA intensity values. HC analysis of the 610 strains in our database revealed that the three clinical isolates from the outbreak did not cluster with the food isolate (Fig. 1A). Analysis of the RMA data scatter plots indicated that the clinical isolates (EC1734, EC1736, and EC1737) are genotypically indistinguishable from one another, while the food isolate (EC1738) was considerably different (Fig. 1B). Our results agree with those of Neil et al. (28) that the food source was never identified. Investigation of the differences between the clinical and food strains revealed an average of 578 probe set differences, 30% of which target regions that are annotated as being prophage encoded.

### (ii) E. coli O157:H7 implicated in the 2006 spinach-associated outbreak.

Most of the clinical isolates were genotypically indistinguishable via the array, with the exception of strains EC4076 and EC4115, which showed signal intensity differences in several prophage-associated probe sets (see Fig. S2 in the supplemental material). Based on high-resolution SNP analysis from WGS data, it has been reported that EC4076 has a prophage deletion and EC4115 is an outlier with strain-specific SNPs and a prophage duplication (29). In addition, Eppinger et al. found that the bovine isolate EC4206 was different from the clinical isolate EC4045 by two strain-specific SNPs (29). Unfortunately, the array probe set intensity data were not able to capture this difference, and EC4206 was genotypically indistinguishable from the clinical isolates.

### (iii) E. coli O104:H4 implicated in the 2011 fenugreek sprout-associated outbreak.

The two clinical isolates from the outbreak that were analyzed are indistinguishable based on the probe set intensity data (see Fig. S3 in the supplemental material). Based on HC and scatter plot analyses, two clinical strains from the Republic of Georgia in 2009 are the nearest neighbors to the German outbreak strain, with similar virulence markers (*stx*_2_, *agg1C*, and *aggR*) and <1% probe set differences. The O104:H4 reference isolate 55989 from the Central African Republic is more distant, with 3% of the total probe sets being different from the German outbreak strain. The probe set percent difference values obtained here for the O104:H4 strain using the FDA-ECID array are similar to those we obtained in a previous study using our FDA-ECSG array (22).

### Evolutionary and phylogenetic classification based on SNP array data.

**FIG 2:**
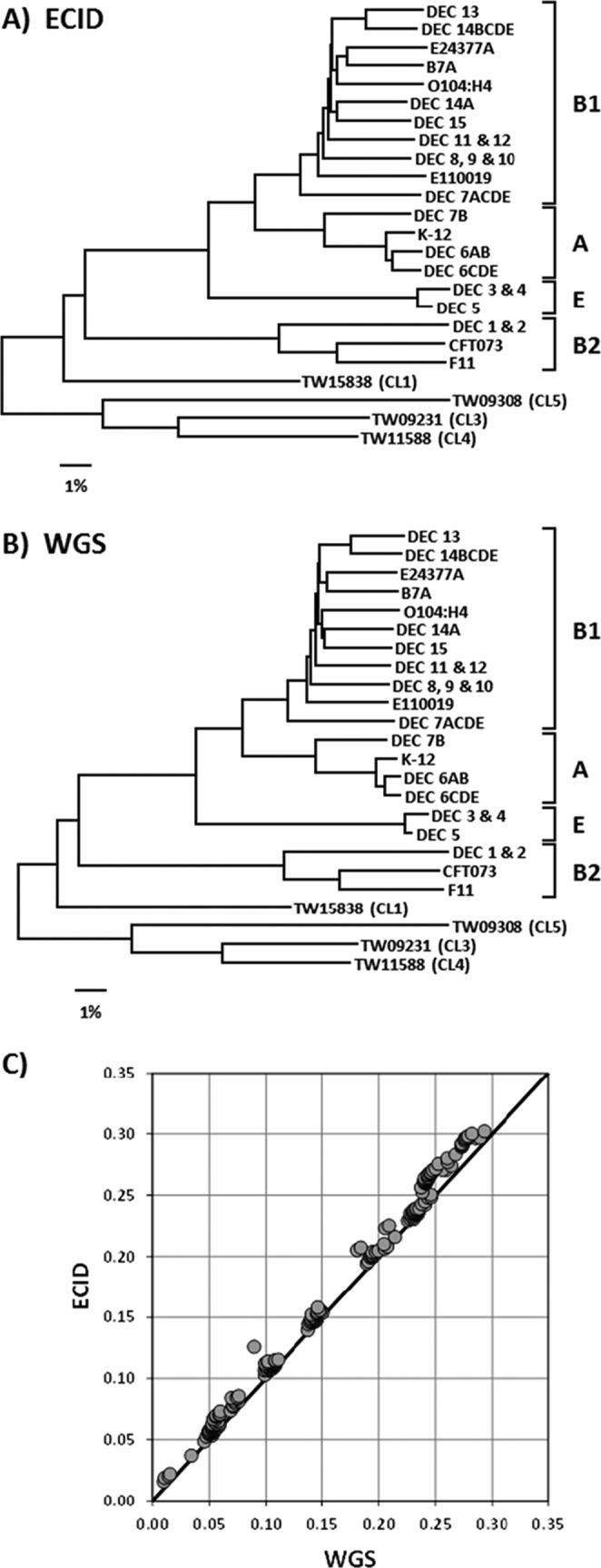
SNP analysis with the FDA-ECID microarray. (A) Neighbor-joining tree constructed using between-group averages for 9,984 SNPs as determined by the FDA-ECID microarray for 103 strains. (B) Neighbor-joining tree constructed using between-group averages from WGS data for the 9,984 SNPs represented on the FDA-ECID microarray for 103 strains. (C) Comparison of the distance matrices used to generate the trees in panels A and B.

**FIG 3:**
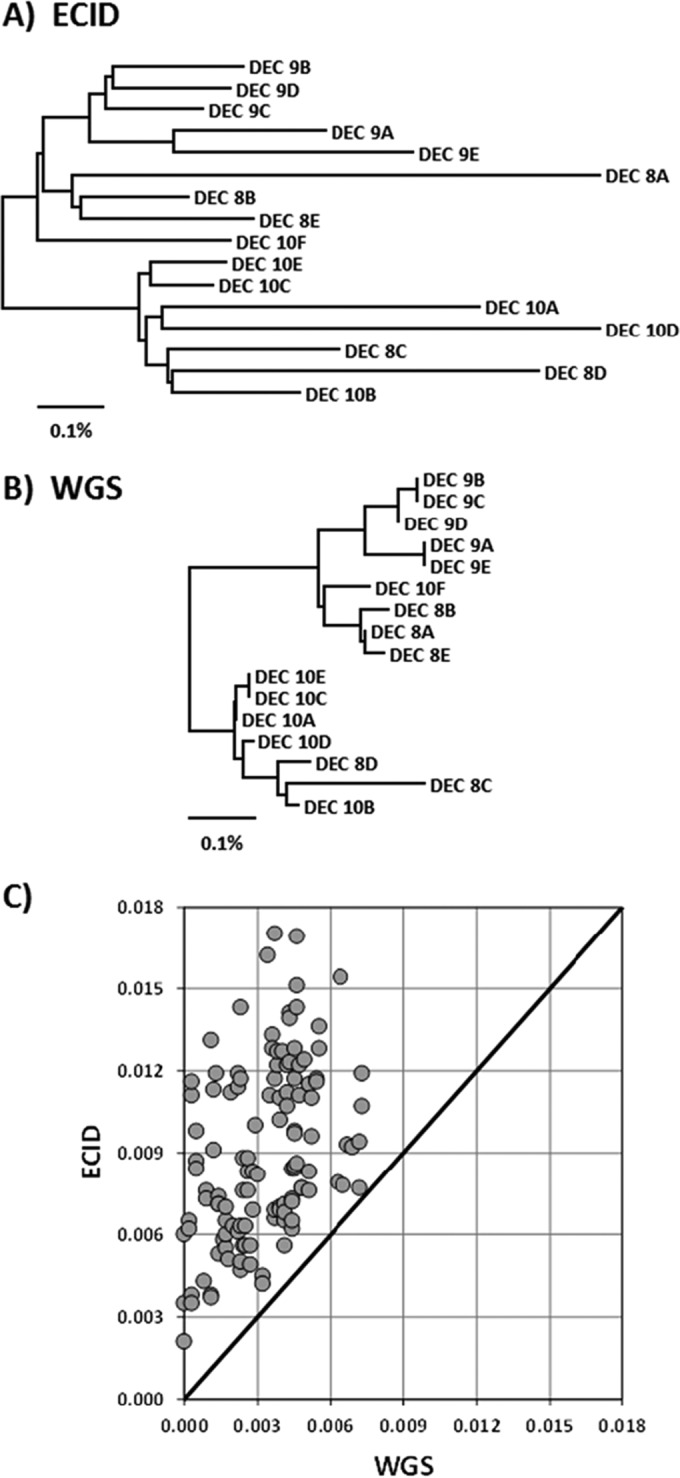
Within-clonal-group SNP analysis with the FDA-ECID microarray. (A) Neighbor-joining tree constructed using 9,984 SNPs as determined by the FDA-ECID microarray for the DEC 8/9/10 strains. (B) Neighbor-joining tree constructed using WGS data for the 9,984 SNPs represented on the FDA-ECID microarray for the DEC 8/9/10 strains. (C) Comparison of the distance matrices used to generate the trees in panels A and B.

In order to more easily view the relationships among the more distinct lineages, the 103 strains were binned into one of 24 groups on the basis of similarities of >99.5% for the WGS data at the 9,984 SNP loci. Phylogenetic trees were then constructed using the average pairwise distances among these 24 groups for both the array SNP data (Fig. 2A) and the corresponding WGS data (Fig. 2B). Both sets of data were capable of distinguishing among the major phylogroups present (A, B1, B2, and E), as well as four of the cryptic lineages of Escherichia. When the underlying distance matrices used to generate the trees were compared, a nearly 1-to-1 linear correspondence was observed, with the data sets being 99.7% correlated (Fig. 2C). However, the array was less effective in recapitulating the relationships among strains within the same clonal group (i.e., those at the >99.5% similarity level). As an example, phylogenetic trees were generated for the DEC 8, 9, and 10 strains (Fig. 3A and B). Branch lengths in the array SNP tree were considerably longer than those in the WGS SNP tree, suggesting errors in the array SNP calls outnumbered the actual SNP differences among the strains. This is supported by the number of variable sites within each data set: 108/9,984 for WGS versus 440/9,984 for the array. Comparison of the distance matrices used to generate the DEC 8/9/10 trees revealed a correlation of 41.5% between the array and WGS data (Fig. 3C). An analysis of the DEC 11 and 12 strains was also performed, with similar results (53 WGS versus 456 array variable sites and 54.4% correlation between data sets) (data not shown). The array and WGS SNP trees for the full 103 strains are available in Fig. S4 and S5 in the supplemental material, respectively.

## DISCUSSION

Upon comprehensive evaluation of 103 strains of E. coli, the FDA-ECID microarray was demonstrated to be a rapid and reliable molecular typing tool capable of differentiating potential disease-causing strains of E. coli from nonpathogenic strains. The array was able to discriminate the strains beyond the level of serotype, including virulence profile and the core genetic backbone. The availability of this depth of information in a single rapid assay is crucial for source attribution and is critical for risk assessment based on the strains' virulence profiles in order to recall implicated foods.

Comparative pan-genome analysis of E. coli pathotypes is rapidly becoming part of many outbreak investigations (30, 31). This level of analysis includes the core genome as well as the distinct genes. Unassembled WGS data in various forms have already been useful for the characterization of strains in outbreaks for E. coli (32). However, to be able to be used as an efficient and high-resolution standard typing method for routine outbreak surveillance, further refinement is necessary. WGS is still laborious and time-consuming and continues to require substantial computer resources as well as bioinformatics training for compilation of useful data for routine surveillance (33, 34). Until the laboratory and bioinformatic limitations with WGS are overcome and it becomes useful as a standard practice, rapid molecular typing methods are still required to provide more information for a strain and species than routine typing.

The FDA-ECID array was designed for molecular serotyping and detection of virulence genes, as well as 41,932 genic regions, enabling the array to provide genome-wide characterization of E. coli strains. The overall time from genomic DNA to analyzed data is less than 24 h, with only 2 h of actual hands-on time required. Current protocols for E. coli detection, isolation, and identification from foods include a combination of bacteriological culture-based enrichments, biochemical, immunological, and molecular methods, and serotyping using specific antisera (35). These methods are time-consuming, have logistical constraints when processing large numbers of samples, and may not be fully reliable due to limited sensitivity.

Molecular-based methods, such as PCR and immunological assays (36, 37), have been used to determine a limited number of E. coli serotypes. The major limitation of using PCR and Luminex-based assays for molecular serogrouping is the presence of over 220 O-antigen types in E. coli and Shigella spp. While other highly parallel molecular serotyping methods are available, they are limited in their number of targets relative to the microarray (500 Luminex targets versus 50,000 Affymetrix targets). Therefore, multiple independent assays would need to be performed for serotyping, virulence, and subtyping.

In a recent study, the significance of accurate identification of O types was noted by showing that the O-antigen cluster-based diversification of E. coli is lineage dependent (38). In combination, certain O and H types are associated with bacterial clones that cause specific kinds of disease (39). Therefore, serotype information can be used for evaluating the disease potential in humans along with virulence marker information during epidemiological outbreak investigations. The array was able to simultaneously assay for 152 O types and identified the O types for 99 of the 103 strains investigated in this study, with the four nontypeable strains due to the lack of representation of their specific O types on the array. In a recent publication (27), we have shown the practical applications of this array for molecular serotyping of STEC isolated from fresh produce. In comparison to traditional serotyping, which may take from a few days to weeks to complete and is limited due to the number of available antisera as well as cross-reactivity issues, the FDA-ECID array was shown to be an effective alternative. The array was able to molecularly serotype produce STEC strains, many of which could not be serotyped or had only partial serotypes based on antisera.

The FDA-ECID array can also provide whole-genome content data and virulence markers for strain characterization which otherwise would need separate genome sequencing or gene-specific PCR assays to determine and confirm the presence or absence of specific virulence genes. Due to the significant variation in virulence content within a serotype, information on virulence markers carried by a strain is equally important. Molecular risk evaluation methods based on assessment of virulence markers have been used to predict if strains of E. coli might pose a significant threat to human health (40, 41). In this study, we showed how the array can be used for epidemiological investigations. For the 2009 O157:H7 cookie dough-associated outbreak, in which 72 cases of illness were reported from 30 states, our array data confirmed that the clinical isolates are distinct from the food isolate. Similarly for the 2006 O157:H7 spinach-associated outbreak, the array is able to distinguish the clinical strain with a prophage variation compared to other clinical strains from the same outbreak. Finally, for the 2011 German O104:H4 outbreak, the array could accurately serotype and provide overall information on the genomic fingerprint as well as the strain's virulence attributes, such as the combination of Shiga toxin and enteroaggregative features. This type of pan-genome analysis is extremely useful for epidemiological investigations.

To study evolutionary relationships of strains, phylogenetic grouping of E. coli has previously been used for classification of commensal strains from pathogenic strains. E. coli has been subdivided into four main phylogenetic groups (1) and three minor groups (3, 42). It has been shown recently that whole-genome phylogenies have different tree topologies from traditional multilocus sequence typing using seven housekeeping loci (18). Whole-genome-scale global analysis of genomic diversity by using either pan-genome data or SNP-level data can help elucidate the mechanisms that drive diversification. Phylogenies based on WGS data have been used to identify SNPs that are used to generate trees to show relationships of strains from an outbreak (43, 44). It has also been shown that a specific phylogenetic background is required for the acquisition of virulence factors located on pathogenicity islands and plasmids (45). Large portions of bacterial genomes are subject to rapid change through chromosome-integrated prophages or the acquisition or loss of plasmids resulting in longer branch lengths in the pan-genomic trees from closely related strains (46).

The genomic positions for the defined set of SNPs on the array were used to extract SNPs from WGS data for reference sequenced strains. The array is capable of detecting whether or not the test strain matches the reference strain at each SNP position. However, if the test strain does not match the reference, the current analysis method does not determine the actual nucleotide, but the SNP positions can be compared to those from other studies. When trees based on the WGS SNPs were compared to the experimental data, we found a 99.7% correlation for the between-group distances, indicating that the array SNPs accurately recapitulate the relationships determined by the WGS SNPs. The time required to run samples and determine the genotypic relationships of strains during an outbreak is significantly less when using a microarray (<24 h) than when performing a 2× 150-bp WGS run yielding 80× coverage (∼48 h). Data processing and bioinformatic challenges are still a bottleneck for WGS data (47, 48), while the relationships between strains can be determined from a typical microarray run in less than an hour, thereby allowing for a rapid genome-scale analysis. Current approaches of sequencing microbial genomes at a high resolution are still relatively expensive and time-consuming. The Affymetrix microarray-based resequencing approach offers an alternative for collecting SNP information. This kind of comparative phylogenomic analysis is important when evaluating potential human health risk criteria.

A limitation of the FDA-ECID microarray in its current configuration is that of the over 220 named O types present within E. coli and Shigella, only the 152 O types with sequence data available when the array was designed are represented. Sequence data for the complete set of reference O types were recently made available, and we are looking into the feasibility of designing a second version of the array with all known O types. Thus, sequence information that was not available and therefore not included at the time of the design will not be able to be detected using the array. Another limitation of the array is that it can accurately and reliably identify all known H types with the exception of H1 and H12, for which discrimination is difficult due to the high degree of homology (>98%) between these alleles. The probe sets are unable to consistently achieve this level of discrimination for variants that differ from the H1 and H12 sequences represented on the array. Similarly, we are unable to accurately discriminate *stx*_2_ allelic subtypes and a few other virulence subtypes due to sequence similarity of the alleles.

In conclusion, the FDA-ECID array is a rationally designed, rapid, and easy-to-use E. coli genomic characterization tool. It is the only method known so far that provides an opportunity to simultaneously test for the presence of 152 O types, 53 H types, and numerous virulence markers in less than 24 h. In addition, the SNP results presented suggest that the phylogeny based on 9,984 SNPs is enough to determine the lineage-dependent diversification of E. coli.

## Supplementary Material

Supplemental material
