# Supplementary material for: FDA Escherichia coli Identification (FDA-ECID) Microarray: a Pangenome Molecular Toolbox for Serotyping, Virulence Profiling, Molecular Epidemiology, and Phylogeny
Source: Appl Environ Microbiol. 2016 May 16;82(11):3384–94. doi: 10.1128/AEM.04077-15 (PMC4959244; doi:10.1128/AEM.04077-15)
Supplement: Supplemental material [file supp_82_11_3384__index.html]

Supplemental material 

# FDA Escherichia coli Identification (FDA-ECID) Microarray: a Pangenome Molecular Toolbox for Serotyping, Virulence Profiling, Molecular Epidemiology, and Phylogeny

## Supplemental material

- Supplemental file 1 -

  Hierarchical cluster analysis of FDA-ECID RMA intensity data for 41,932 probe sets (Fig. S1), FDA-ECID RMA scatter plots of the 2006 spinach-associated outbreak reference isolate EC4045 (Fig. S2), hierarchical cluster and RMA scatter plots of O104:H4 isolates (Fig. S3), and phylogenetic relationships of 103 strains based on FDA-ECID microarray SNP data (Fig. S4) and WGS data for the FDA-ECID SNP sites (Fig. S5).

  PDF, 1.4M
- Supplemental file 2 -

  Strains run so far and their molecular serotypes determined by the array (Table S1), information on the array design (Table S2), information on Shiga toxin probe sets (Table S3), information on intimin probe sets (Table S4), and information on the SNP positions (Table S5).

  XLSX, 262K
