# Supplementary material for: FDA Escherichia coli Identification (FDA-ECID) Microarray: a Pangenome Molecular Toolbox for Serotyping, Virulence Profiling, Molecular Epidemiology, and Phylogeny
Source: Appl Environ Microbiol. 2016 May 16;82(11):3384–94. doi: 10.1128/AEM.04077-15 (PMC4959244; doi:10.1128/AEM.04077-15)

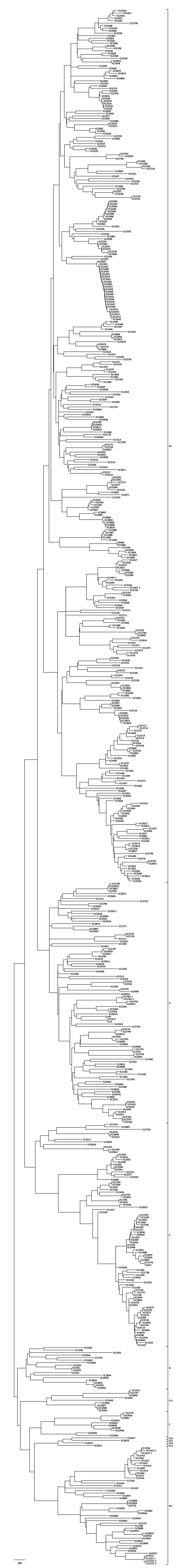

Supplemental Figure 1. Hierarchical cluster analysis of FDA-ECID RMA intensity data for 41,932 probesets. This dendrogram was generated using the number of probesets that were greater than 3-fold different in 610 isolates. Phylogroups are indicated by the square brackets. The scale bar represents the number of probeset differences.

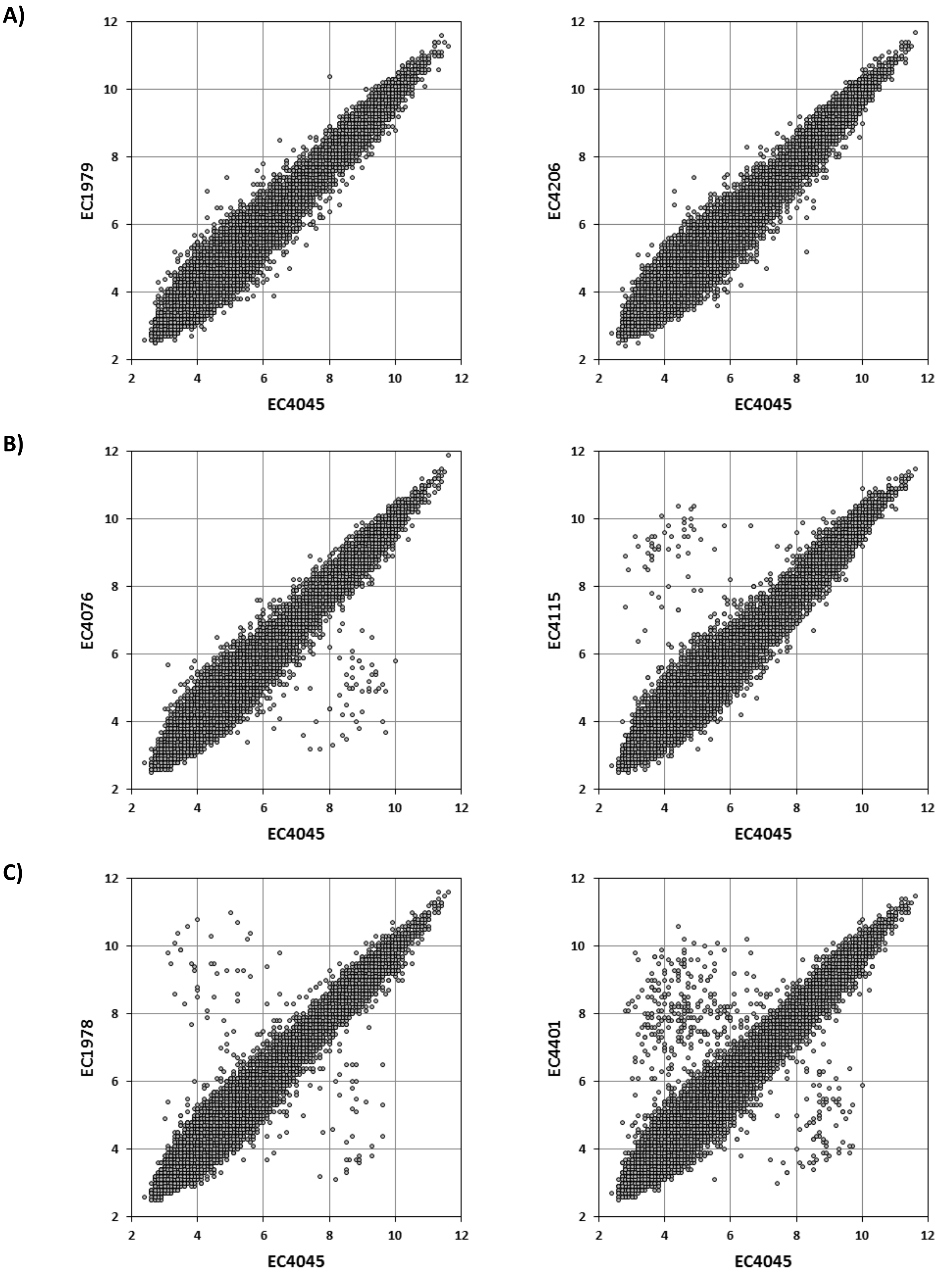

Supplemental Figure 2. FDA-ECID RMA scatter plots of the 2006 spinach-associated outbreak reference isolate EC4045. A) Comparisons to two isolates that are genotypically indistinguishable from EC4045: clinical isolate EC1979 and bovine isolate EC4206. B) Comparisons to two outbreak-associated variants: clinical isolate EC4076 and clinical outlier EC4115. C) Comparisons to two closely related isolates not associated with the outbreak: EC1978, a bovine isolate from 2012, and EC4401, a clinical isolate from a 2006 lettuce-associated outbreak.

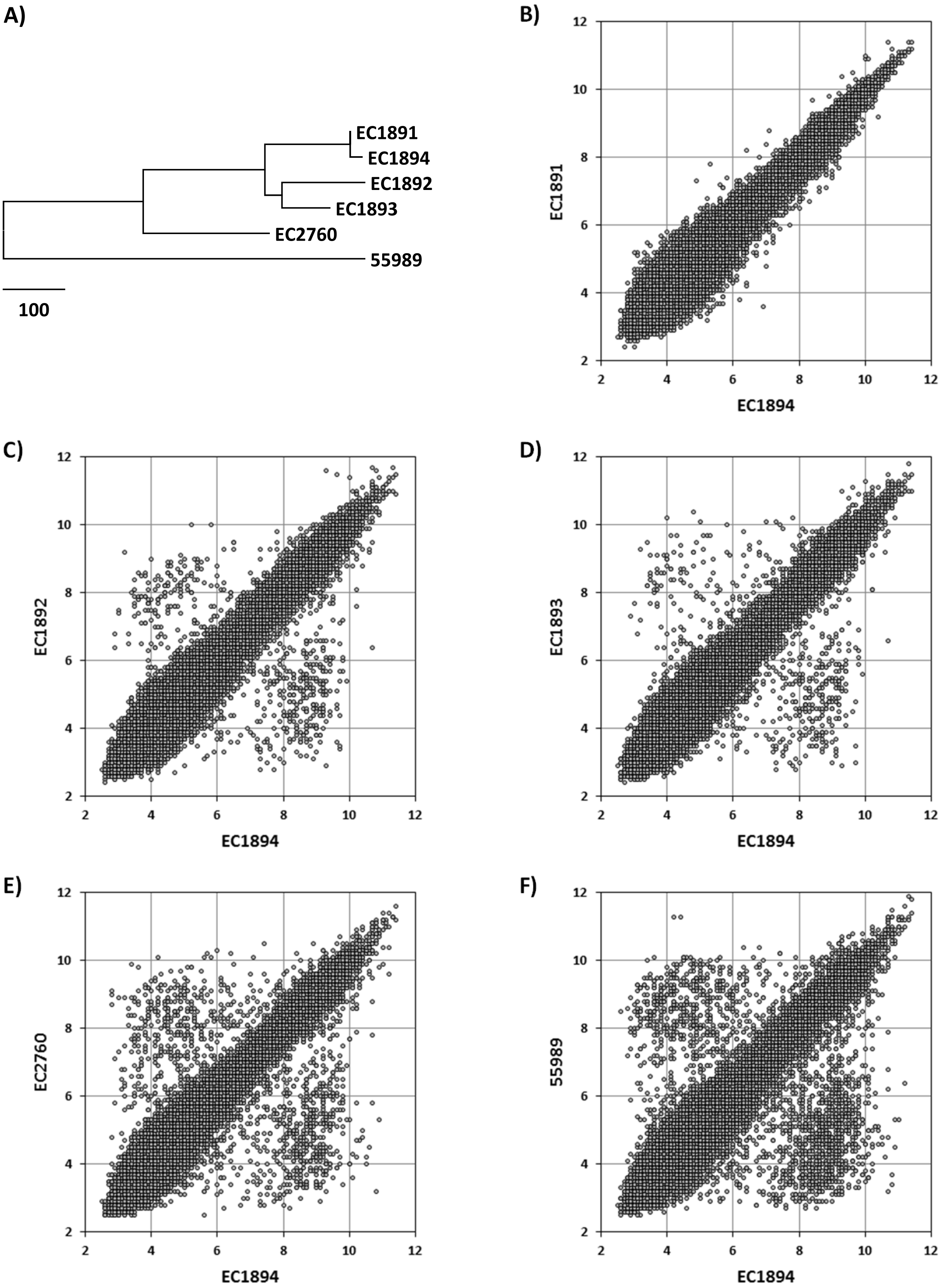

Supplemental Figure 3. Hierarchical cluster and RMA scatter plots of O104:H4 isolates. A) Dendrogram of O104:H4 strains analyzed by the FDA-ECID array. Scale bar represents 100 probeset differences. B) Comparison of two 2011 sprout-associated outbreak clinical isolates. C & D) Comparisons of the 2011 outbreak isolate to two Republic of Georgia isolates from 2009. E & F) Comparisons of the 2011 outbreak isolate to two more distantly related isolates: EC2760 from Denmark in 2000 and 55989 from the Central African Republic in 2002.

Supplemental Figure 4. Phylogenetic relationships of 103 strains based on FDA-ECID microarray SNP data. This neighbor joining tree was constructed using data for 9984 SNP locations as determined by the FDA-ECID microarray.

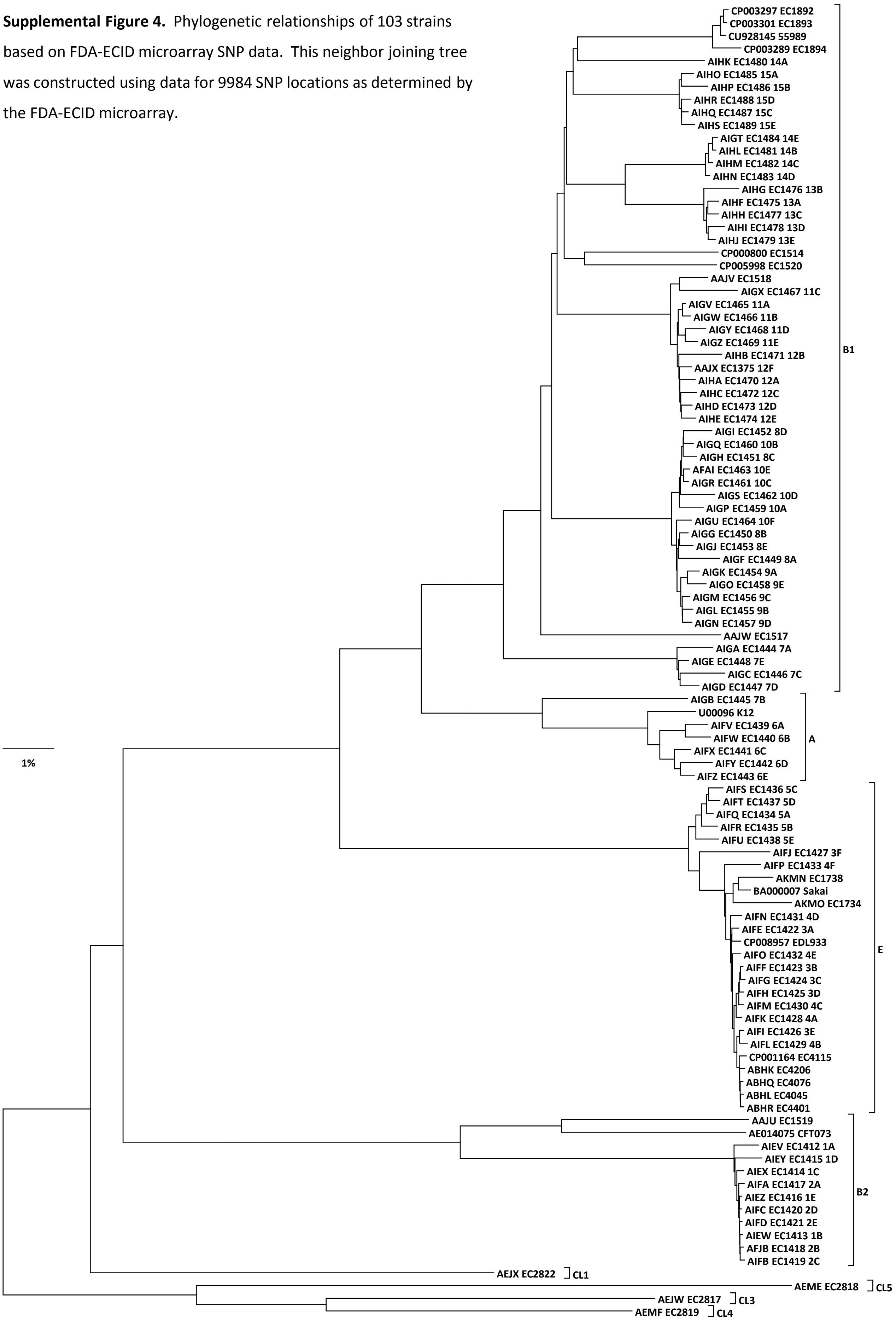

Supplemental Figure 5. Phylogenetic relationships of 103 strains based on WGS data for the FDA-ECID SNP sites. This neighbor joining tree was constructed using the WGS data for the 9984 SNP locations selected for inclusion on the FDA-ECID microarray.

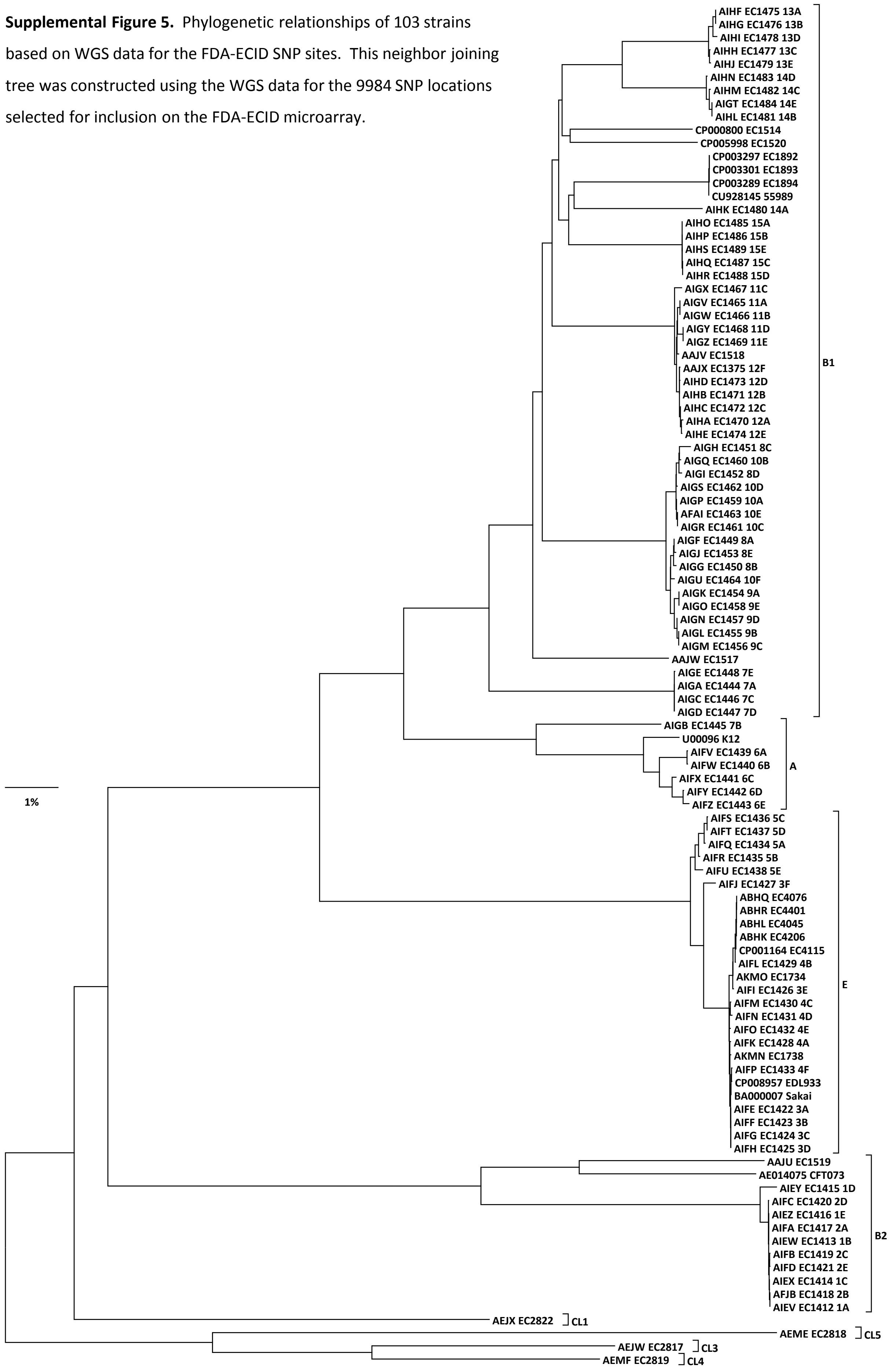

Supplement: Supplemental material [file AEM.04077-15_zam999117173so1.pdf]
